# The CaMK Family Differentially Promotes Necroptosis and Mouse Cardiac Graft Injury and Rejection

**DOI:** 10.3390/ijms25084428

**Published:** 2024-04-17

**Authors:** Haitao Lu, Jifu Jiang, Jeffery Min, Xuyan Huang, Patrick McLeod, Weihua Liu, Aaron Haig, Lakshman Gunaratnam, Anthony M. Jevnikar, Zhu-Xu Zhang

**Affiliations:** 1 Matthew Mailing Centre for Translational Transplantation Studies, London Health Sciences Centre, London, ON N6A 5A5, Canada; hlu273@uwo.ca (H.L.); jevnikar@uwo.ca (A.M.J.); 2 Department of Pathology, Western University, London, ON N6A 3K7, Canada; 3 Department of Microbiology and Immunology, Western University, London, ON N6A 3K7, Canada; 4 Multi-Organ Transplant Program, London Health Sciences Centre, London, ON N6A 5A5, Canada; 5 Division of Nephrology, Department of Medicine, Western University, London, ON N6A 3K7, Canada

**Keywords:** CaMK, Drp1, PGAM5, necroptosis, transplantation, heart

## Abstract

Organ transplantation is associated with various forms of programmed cell death which can accelerate transplant injury and rejection. Targeting cell death in donor organs may represent a novel strategy for preventing allograft injury. We have previously demonstrated that necroptosis plays a key role in promoting transplant injury. Recently, we have found that mitochondria function is linked to necroptosis. However, it remains unknown how necroptosis signaling pathways regulate mitochondrial function during necroptosis. In this study, we investigated the receptor-interacting protein kinase 3 (RIPK3) mediated mitochondrial dysfunction and necroptosis. We demonstrate that the calmodulin-dependent protein kinase (CaMK) family members CaMK1, 2, and 4 form a complex with RIPK3 in mouse cardiac endothelial cells, to promote trans-phosphorylation during necroptosis. CaMK1 and 4 directly activated the dynamin-related protein-1 (Drp1), while CaMK2 indirectly activated Drp1 via the phosphoglycerate mutase 5 (PGAM5). The inhibition of CaMKs restored mitochondrial function and effectively prevented endothelial cell death. CaMKs inhibition inhibited activation of CaMKs and Drp1, and cell death and heart tissue injury (n = 6/group, *p* < 0.01) in a murine model of cardiac transplantation. Importantly, the inhibition of CaMKs greatly prolonged heart graft survival (n = 8/group, *p* < 0.01). In conclusion, CaMK family members orchestrate cell death in two different pathways and may be potential therapeutic targets in preventing cell death and transplant injury.

## 1. Introduction

Inflammation in transplantation directly results in diverse cell death programs, including necrosis and apoptosis, which promote transplant injury and rejection. Necrosis promotes cellular swelling, plasma membrane rupture, and the release of pro-inflammatory molecules which worsen inflammation [1,2,3]. Necrosis is also regulated, and the most studied form, necroptosis, is initiated by the receptor-interacting protein kinase family (RIPK1 and 3) and mixed lineage kinase domain like protein (MLKL) [1,2,3,4].

Other intracellular signaling mechanisms of necroptosis are likely to exist. Mitochondria play a major role in cell metabolism and participate in cell death. Whether mitochondria are directly involved in programmed forms of necrosis remains controversial [5,6,7] and contradictory data suggest that both mitochondrial-dependent and -independent forms of necrosis or necroptosis participate [5,6,7,8].

We have previously demonstrated that RIPK3-mediated necroptosis in donor heart grafts can promote inflammatory injury and transplant rejection [9,10]. The elimination of RIPK3 expression in mouse microvascular endothelial cells (MVEC) or donor allografts attenuated cell and tissue necrosis, and reduced rejection [9,10]. Furthermore, we found that mitochondrial membrane permeability is an important mechanistic mediator of necroptosis in MVEC, suggesting that blocking this process might prevent cardiac transplant ischemia injury and rejection [11,12]. Understanding the relation of RIPK activation to mitochondrial damage during necroptosis would be important in developing strategies that target cell death and transplant rejection. In this study, we found that the calmodulin-dependent protein kinase (CaMK) family plays a crucial role in necroptosis and that RIPK3, and CaMK1, 2, and 4 form a ‘death complex’. CaMK2 activates phosphoglycerate mutase 5 (PGAM5) which in turn activates mitochondrial dynamin-related protein-1(Drp1), while CaMK1/4 directly activate Drp1. Importantly, inhibition of CaMK function attenuated cell death and significantly prolonged murine heart transplant survival.

## 2. Results

### 2.1. The CaMK Family Participates in MVEC Necroptosis

**Figure 1:**
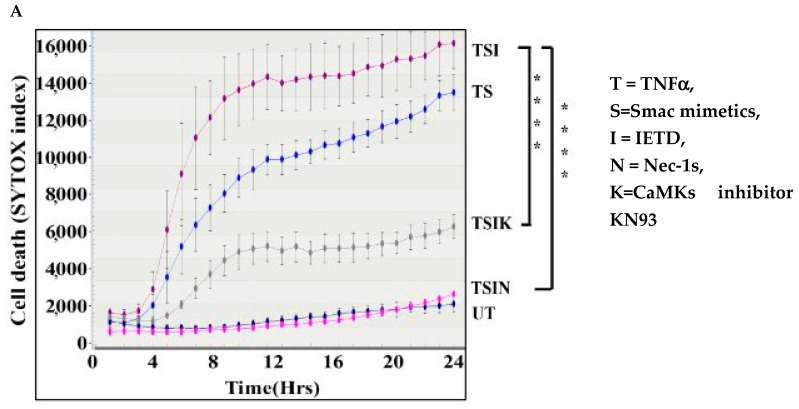

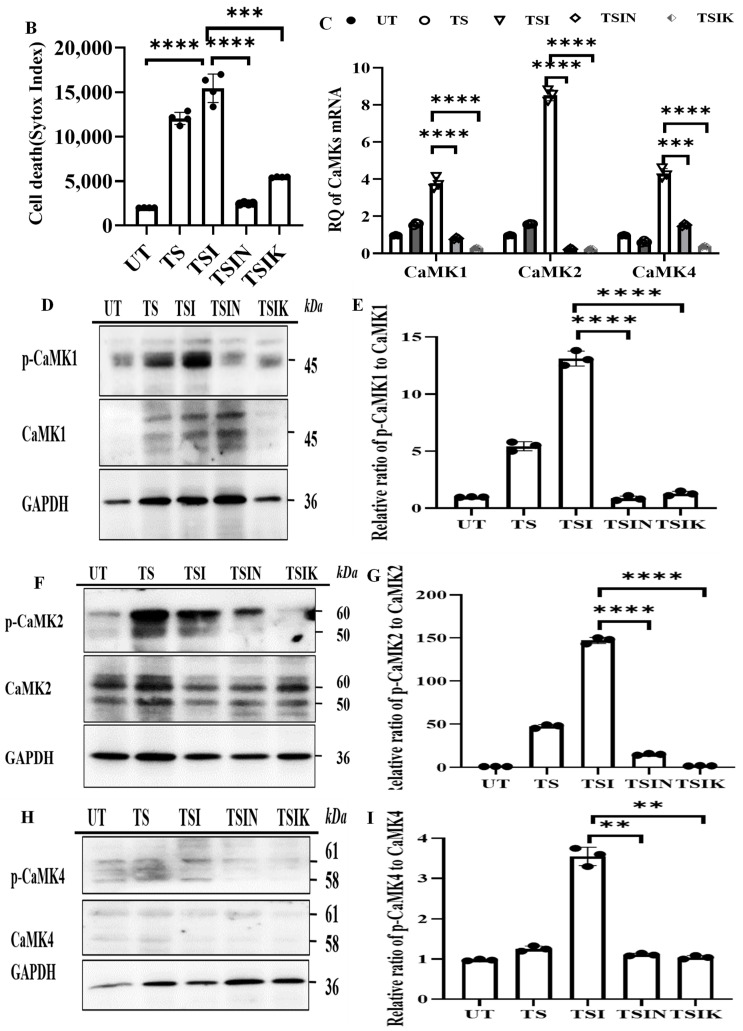
The CaMK family participates in MVEC necroptosis. (**A**) Cells (20 × 10^3^/well) were seeded in quadruplicates in a 96-well plate. Cell death was induced by TNFα (T, 20 ng/mL) with Smac mimetic BV6 (S, 2 μM). Apoptosis was inhibited by caspase-8 inhibitor z-IETD (I, 30 μM). Necroptosis was inhibited by RIPK1 inhibitor Nec-1s (N, 10 μM). CaMK was inhibited by KN93 (K, 20 μg/mL). Cell death was detected by SYTOX Green uptake into the dead cell from 0 to 24 h by IncuCyte Image system (Essen Bioscience, Ann Arbor, MI, USA). (**B**) SYTOX uptake was quantified at 24 h. Data are shown as mean ± standard deviation (SD) of quadruplicates and representative of three independent experiments. **** *p* < 0.0001. *t*-test. (**C**) Expression of CaMK1, CaMK2, and CaMK4 was quantified by real time PCR after cell death induction for 4 h. β-actin was used as endogenous control for mRNA expression. Data are shown as mean ± SD of three independent experiments. Western blot analysis of CaMK1 and p-CaMK1 (**D**,**E**), CaMK2 and p-CaMK2 (**F**,**G**), and CaMK4 and p-CaMK4 (**H**,**I**). Cells were collected for Western blot analysis 5 h after cell death induction. GAPDH was used as loading control. Images were quantified by densitometry (ImageJ 1.54g). Relative ratio = phosphorylated protein/total protein. Data are shown as the mean ± SD of three independent experiments. ** *p* < 0.01, *** *p* ≤ 0.001, **** *p* ≤ 0.0001; 1-way ANOVA; Tukey’s multiple comparisons.

We sought to determine upstream mechanisms of mitochondrial damage and MVEC death to identify any effective target(s) to control cell death. Exposure to TNFα induced death. The addition of the caspase-8 inhibitor IETD failed to block cell death (Figure 1A,B), supporting that caspase-8-independent cell death is due to necroptosis [9,10]. The observed TNFα-induced cell death was inhibited by the addition of RIPK1 inhibitor necrostatin-1s (Nec-1s, Figure 1A,B), confirming that it was indeed due to necroptosis. Interestingly, we found that the addition of the CaMKs inhibitor KN93 significantly reduced cell death (Figure 1A,B), implying that CaMKs play a role in necroptosis. In addition, we found that CaMK1, 2, and 4 mRNA expression was significantly increased during necroptosis (Figure 1C). Furthermore, phosphorylated CaMK1–4 proteins were increased during induction of necroptosis, respectively (Figure 1D–I collectively suggesting that CaMKs may participate in necroptosis).

**Figure 2:**
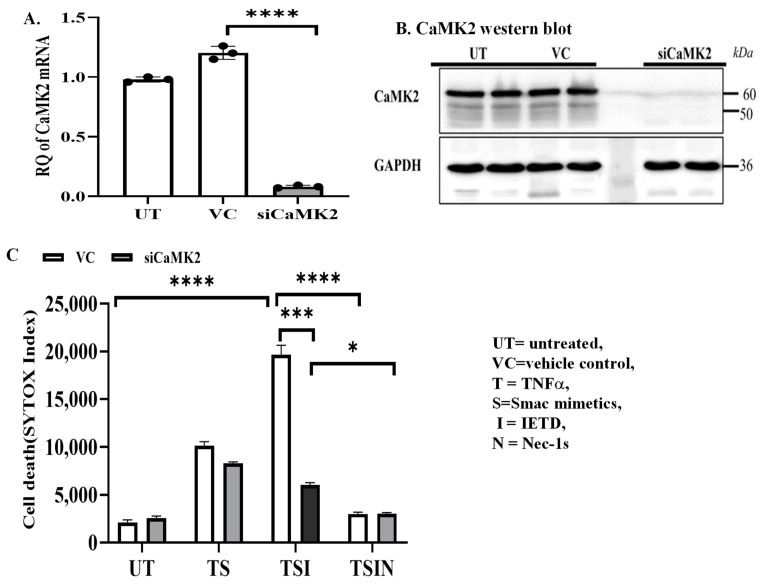

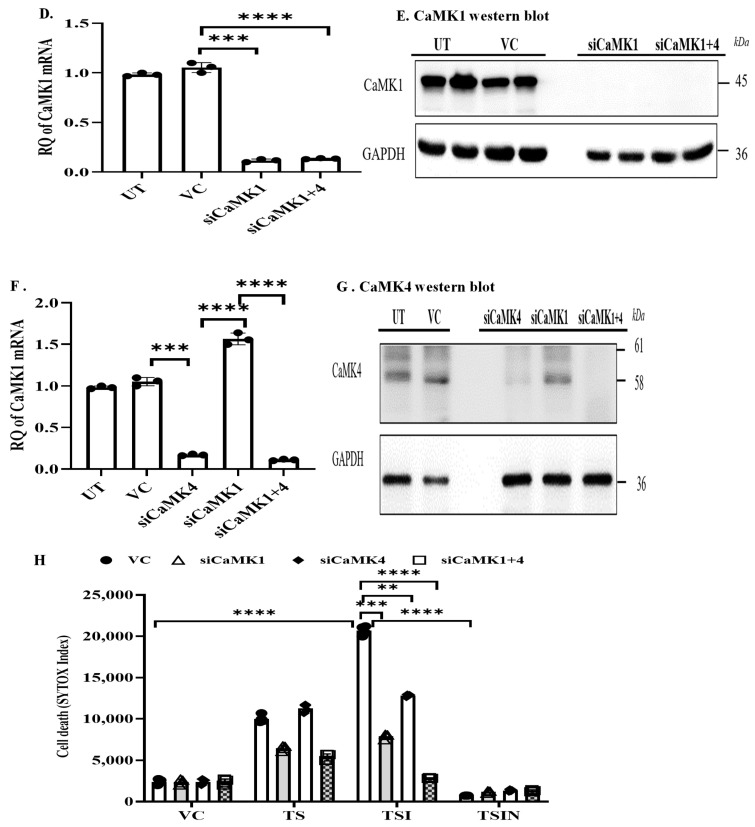
CaMKs participate in necroptosis. CaMK2δ silencing in MVECs was confirmed by PCR 16 h after siRNA treatment (**A**) and Western blot (**B**) 24 h after siRNA treatment. Untreated cells (UT) or vehicle control (VC, transfection reagent) treated cells were used as controls. GAPDH from the same blot was used as the loading control. Data are pooled and represent three independent experiments. (**C**) CaMK2δ-siRNA or VC treated cells were harvested after 24 h and subjected to the cell death assay. SYTOX uptake/cell death was monitored by IncuCyte Image system. Data are shown as mean ± SD of quadruplicates and represent three independent experiments. siRNA-induced silencing of CaMK1 (**D**,**E**) and CaMK4 (**F**,**G**) in MVECs was confirmed by PCR and Western blot analysis. (**H**) CaMK1, CaMK4, or CaMK1+4 siRNAs or vehicle control (VC, EndoFectin) treated cells were harvested after 24 h and subjected to the cell death assay. SYTOX uptake was monitored for 24 h by IncuCyte Image system. Data are shown as mean ± SD of quadruplicates at 24 h and represent three independent experiments. * *p* ≤ 0.05, ** *p*≤0.01, *** *p* ≤ 0.001, **** *p* ≤ 0.0001; 1-way ANOVA; Tukey’s multiple comparisons.

Next, we examined the role of each member of the CaMK family in MVEC necroptosis, respectively. CaMK2 was efficiently silenced by siRNA as confirmed by PCR analysis and Western blot analysis (Figure 2A,B). Necroptosis was decreased in the CaMK2 silenced MVECs compared to the control (Figure 2C), suggesting CaMK2 participates in necroptosis. We then silenced the expression of CaMK1 and CaMK4 in MVECs using siRNA (Figure 2D–G). Cell death was significantly attenuated upon silencing of either CaMK1 or CaMK4 MVECs when compared to their respective controls (Figure 2H). However, the effect on inhibition of necroptosis was additive when both CaMK1 and 4 were silenced (Figure 2H). Hence, our data suggest that CaMK family members participate in MVEC necroptosis that is triggered by TNFα.

### 2.2. RIPK3 and the CaMK Family Members Form a Complex in Necroptosis

**Figure 3:**
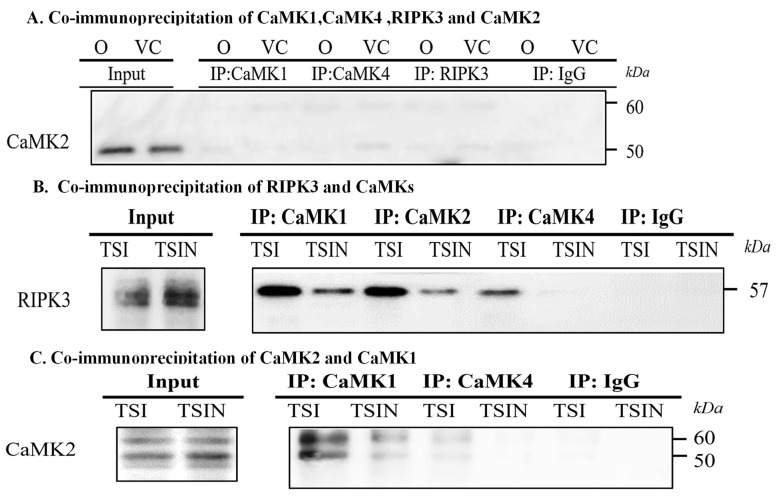

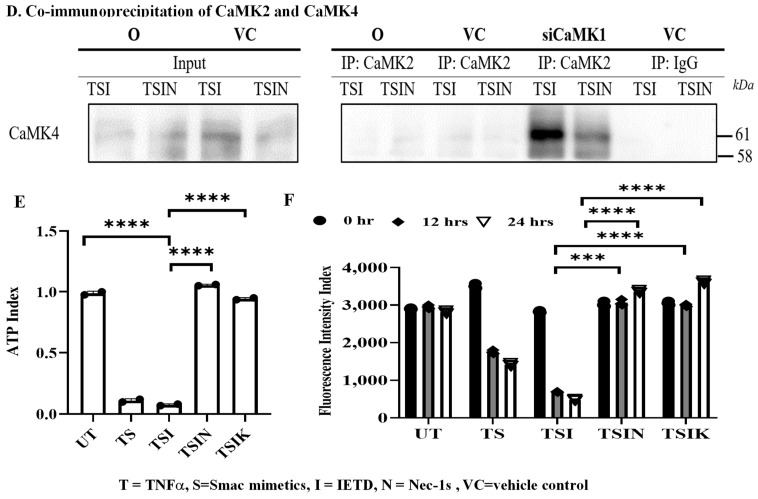
RIPK3 and CaMKs form a complex during necroptosis. Cells were induced to undergo necroptosis, as shown in Figure 1, and collected after 4 h. (**A**) Untreated and vehicle control treated cells were used as co-immunoprecipitation controls. Cell lysates were immunoprecipitated with CaMK1, CaMK4, and RIPK3 antibodies, respectively, and followed by Western blot analysis to detect CaMK2. (**B**) Cell lysates were immunoprecipitated with CaMK1, CaMK2, and CaMK4 antibodies, respectively. Rabbit IgG were used as control. The immunoprecipitants were used to detect RIPK3 in Western blot analysis. (**C**) Cell lysates were immunoprecipitated with CaMK1 and CaMK4 antibodies. The immunoprecipitants were used to detect CaMK2 in Western blot analysis. (**D**) CaMK1 siRNA or vehicle treated cells were harvested after 24 h for cell death induction and then collected after 4 h. Cell lysates were immunoprecipitated with CaMK2 antibody or rabbit IgG. The immunoprecipitants were used to detect CaMK4 in Western blot analysis. Data (**B**–**D**) represent three independent experiments. (**E**) Cell death was induced as described in Figure 1. ATP level was quantified by the CellTiter-Glo^®^ Luminescent Cell Viability kit. Data are shown as mean ± SD of three independent experiments. (**F**) Mitochondria were probed by MitoTracker. Fluorescent intensity was automatically quantified by the IncuCyte System. Data are shown as mean ± SD of three independent experiments. *** *p* ≤ 0.001; **** *p* ≤ 0.0001, 1-way ANOVA; Tukey’s multiple comparisons.

Firstly, we wanted to confirm if RIPK3 interaction is upstream of CaMKs by using co-immunoprecipitation analyses. In untreated and vehicle control treated cells, CaMK2 was not co-immunoprecipitated with CaMK1, CaMK4 and RIPK3 (Figure 3A). However, under necroptosis conditions, RIPK3 was co-immunoprecipitated with CaMK1, CaMK2, and CaMK4, respectively (Figure 3B), suggesting that RIPK3 is linked to CaMKs. As shown in Figure 3C, CaMK1 was co-immunoprecipitated with CaMK2. However, only a low level of CaMK4 was co-immunoprecipitated with CaMK2 (Figure 3C). Interestingly, when CaMK1 was silenced in MVECs, more CaMK4 protein was co-immunoprecipitated with CaMK2 (Figure 3D), suggesting that CaMK4 may play a complementary role in cell death, when CaMK1 is reduced or absent. Taken together, these studies suggest RIPK3 and CaMKs form a complex, resulting in mutual activation during induction of cell death.

To test if mitochondrial dysfunction is a down-stream effect of CaMKs, we measured ATP levels during cell death. ATP levels were substantially decreased during necroptosis, whereas inhibition of CaMKs by KN93 or inhibition of necroptosis by Nec-1s restored ATP production (Figure 3E). In addition, the fluorescent intensity of mitochondria was substantially decreased during necroptosis, which could be recovered by adding KN93 or Nec-1s (Figure 3F). These data suggest that mitochondrial dysfunction is a down-stream effect of CaMKs. We next aimed to identify the mitochondrial signaling molecules down-stream of CaMKs.

### 2.3. CaMK1 and CaMK4 Directly Bind to and Phosphorylate Drp1 while CaMK2 Indirectly Regulates Drp1 Phosphorylation via PGAM5

Drp1 is an important molecule for inducing mitochondrial fragmentation and dysfunction through excessive fission [13]. During mitochondrial fission, high Ca2^+^ intake and a lower ATP level may be associated with the function of CaMKs family proteins. Thus, we tested whether CaMKs and PGAM5 and/or Drp1 interact with each other.

**Figure 4:**
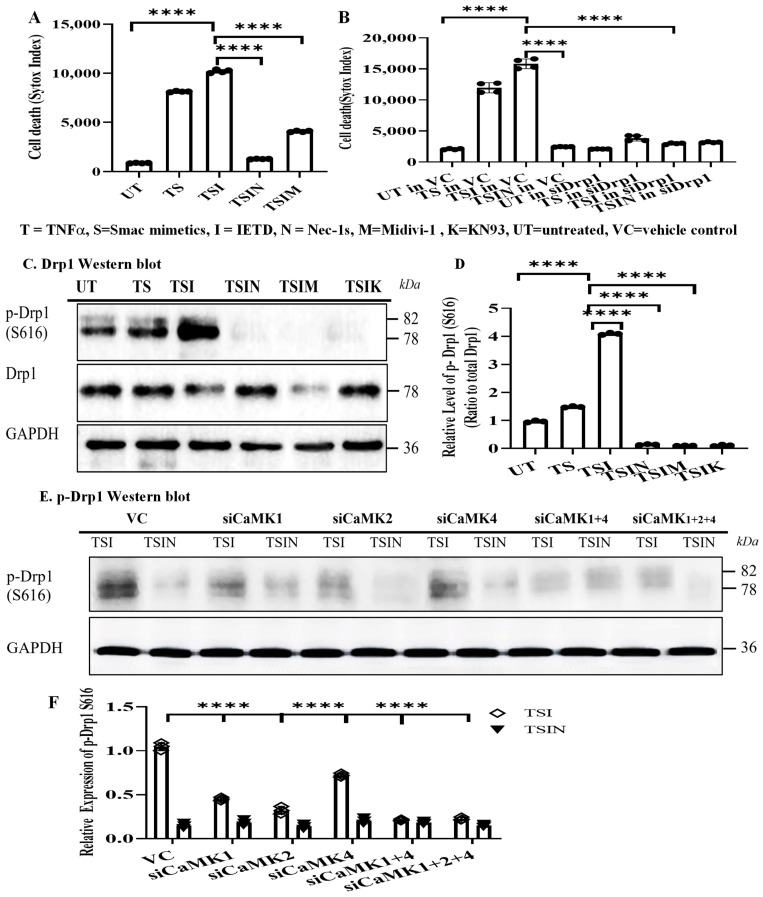
CaMKs are responsible for Drp1 activation. (**A**) Cell death was induced as described in Figure 1. Drp1 inhibitor Midivi-1 (50 μM) was added. Cell death was detected by IncuCyte Image system. SYTOX uptake was quantified at 24 h. Data are shown as mean ± SD of quadruplicates and represent three independent experiments. (**B**) Drp1siRNA or vehicle-treated cells were harvested after 24 h and subjected to the cell death assay. Data are shown as mean ± SD of quadruplicates and represent three independent experiments. **** *p* ≤ 0.0001, 1-way ANOVA; Tukey’s multiple comparisons. (**C**) p-Drp1 Western blot. Cell death was induced as described in Figure 1. Drp1 inhibitor Midivi-1 or CaMKs inhibitor KN93 was added. Cells were collected for Western blot analysis 4 h after cell death induction. Untreated (UT) cells were used as controls. (**D**) Images were quantified by ImageJ. Relative Ratio of protein level = p-Drp1/Total Drp1. Data are shown as mean ± SD of three independent experiments. (**E**) CaMK1, CaMK2, CaMK4, CaMK1+4, and CaKM1+2+4 siRNAs-treated cells were harvested after 24 h for the cell death assay. Cells were collected after 4 h for Western blot analysis of p-Drp1 (S616). (**F**) Images were quantified by ImageJ. GAPDH was used to normalize protein levels. Data are shown as mean ± SD of three independent experiments. **** *p* ≤ 0.0001; 1-way ANOVA; Tukey’s multiple comparisons.

Interestingly, the addition of the Drp1 inhibitor, Midivi-1, inhibited necroptosis (Figure 4A). Similarly, silencing Drp1 with siRNA prevented cell necroptosis, suggesting that Drp1 is required for the necroptosis we observed (Figure 4B). Next, we analyzed the mechanism(s) of CaMK-mediated Drp1 activation. The activated (phosphorylated) form of Drp1 (p-Drp1 S616) was inhibited by RIPK1 inhibitor Nec-1s, Drp1 inhibitor Midivi-1, or CaMKs inhibitor KN93 (Figure 4C,D), confirming the role of Drp1 in necroptosis. Furthermore, silencing CaMK1, 2, 4, or CaMK1+2+4 inhibited the level of p-Drp1 (S616) (Figure 4E,F). These results suggested that CaMKs are responsible for Drp1 activation.

**Figure 5:**
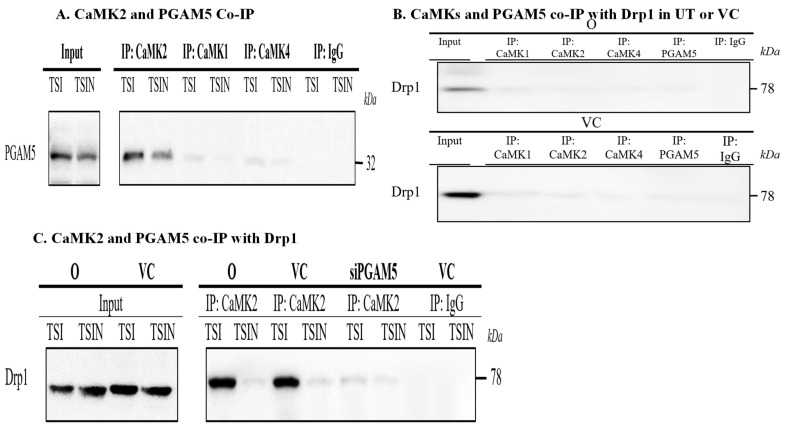

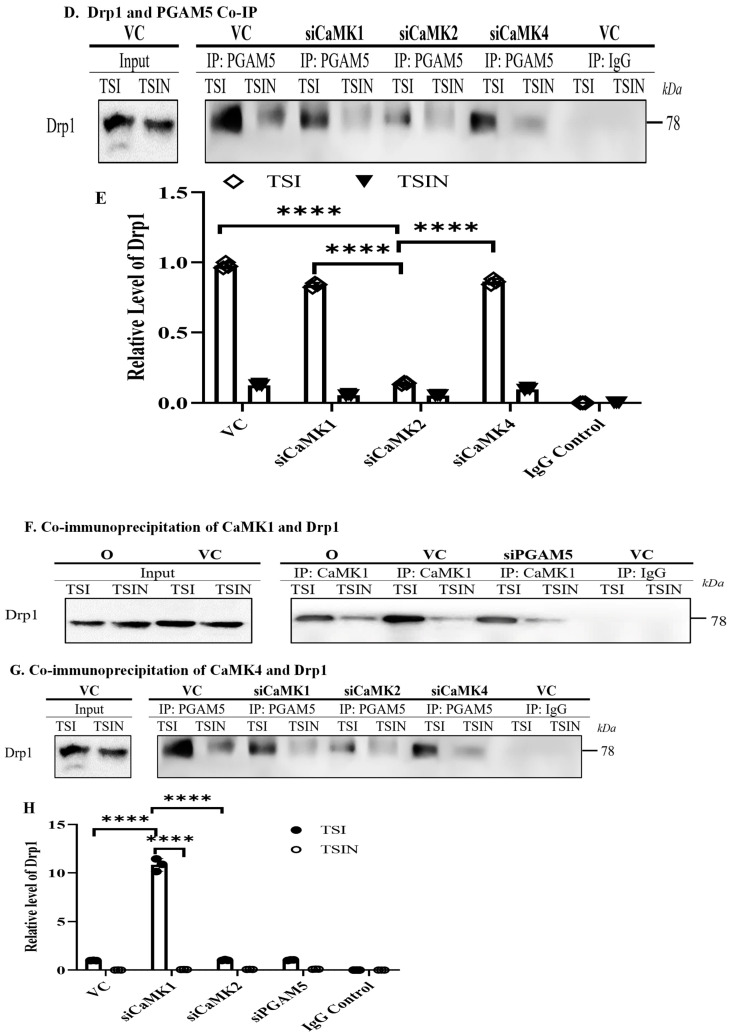
CaMK2 indirectly binds to Drp1 via PGAM5 while CaMK1 and CaMK4 directly bind to Drp1 without PGAM5. (**A**) Cells death was induced as Figure 1 and cells were collected 4 h after. Cell lysates were immunoprecipitated with CaMK1, CaMK2, and CaMK4 antibodies. Rabbit IgG was used as isotype control. The immunoprecipitants were used to detect PGAM5 in Western blot analysis. (**B**) Untreated and vehicle control treated cells were used as co-immunoprecipitation controls. Cell lysates were immunoprecipitated with CaMK1, CaMK2, CaMK4 and PGAM5 antibodies, respectively, and followed by Western blot analysis to detect Drp1. (**C**) PGAM5 siRNA- or vehicle control (VC)-treated cells were harvested after 24 h for the cell death assay. Four hours after, cell lysates were immunoprecipitated with anti-CaMK2 or control IgG. The immunoprecipitants were used to detect Drp1 in Western blot analysis. (**D**) CaMKs siRNAs- or vehicle control-treated cells were harvested after 24 h and used in the cell death assay. Cells were collected after 4 h and cell lysates were immunoprecipitated with PGAM5 antibody or rabbit IgG. Immunoprecipitants were used to detect Drp1 in Western blot. (**E**) Images were quantified by ImageJ. The relative level of Drp1 was calculated against the Drp1 level of necroptotic cells (TSI treated) in vehicle control (VC) group. Data are shown as mean ± SD of three independent experiments. **** *p* ≤ 0.0001; *t*-test. (**F**) PGAM5 siRNA- or VC-treated cells were harvested after 24 h and used in the cell death assay. Cells were collected after 4 h and cell lysates were immunoprecipitated with CaMK1 antibody or control IgG. The immunoprecipitants were used for anti-Drp1 detection in Western blot analysis. (**G**) CaMK1, CaMK2, or PGAM5 siRNA-treated cells were harvested after 24 h for the cell death assay. Cells were collected after 4 h and immunoprecipitated with CaMK4 antibody or control IgG. The immunoprecipitants were used for anti-Drp1 detection in Western blot analysis. (**H**) Images were quantified by ImageJ. The relative level of Drp1 was calculated against the Drp1 level of necroptotic cells (TSI treated) in vehicle control (VC) group. Data are shown as mean ± SD of three independent experiments. **** *p* ≤ 0.0001; 1-way ANOVA; Tukey’s multiple comparisons.

Next, we determined if PGAM5 is required for CaMKs-mediated Drp1 phosphorylation. Interestingly, CaMK2, but not CaMK1 and CaMK4, was co-immunoprecipitated with PGAM5 (Figure 5A), suggesting CaMK2-PGAM5-Drp1 associated in a trimolecular complex. In untreated and vehicle control treated cells, Drp1 was not co-immunoprecipitated with CaMK1, CaMK2, CaMK4 and PGAM5 (Figure 5B). However, under necroptosis conditions, silencing PGAM5 by siRNA abrogated co-immunoprecipitation of CaMK2 and Drp1 (Figure 5C). Furthermore, silencing CaMK2, but not CaMK1 or CaMK4, inhibited the co-immunoprecipitation of PGAM5 and Drp1 (Figure 5D,E), confirming the CaMK2-PGAM5-Drp1 axis in necroptosis.

Next, we analyzed the interaction between CaMK1/CaMK4 and Drp1. The physical interaction between CaMK1 with Drp1 was confirmed by co-immunoprecipitation during necroptosis (Figure 5F). Interestingly, silencing PGAM5 did not prevent CaMK1 binding to Drp1 (Figure 5F), suggesting CaMK1 directly interacts with Drp1 without PGAM5. The interaction between CaMK4 with Drp1 was noted under CaMK1 ‘deficiency’ conditions (Figure 5G,H), suggesting that CaMK4 plays a complementary role for CaMK1. Taken together, our study demonstrated that CaMK1 and CaMK4 can directly mediate Drp1 activation while CaMK2 indirectly regulates Drp1 activation via PGAM5. Hence, CaMKs may be considered as potential therapeutic targets to prevent cell death and graft injury.

### 2.4. Inhibition of CaMKs Attenuated Heart Transplant Injury and Rejection

**Figure 6:**
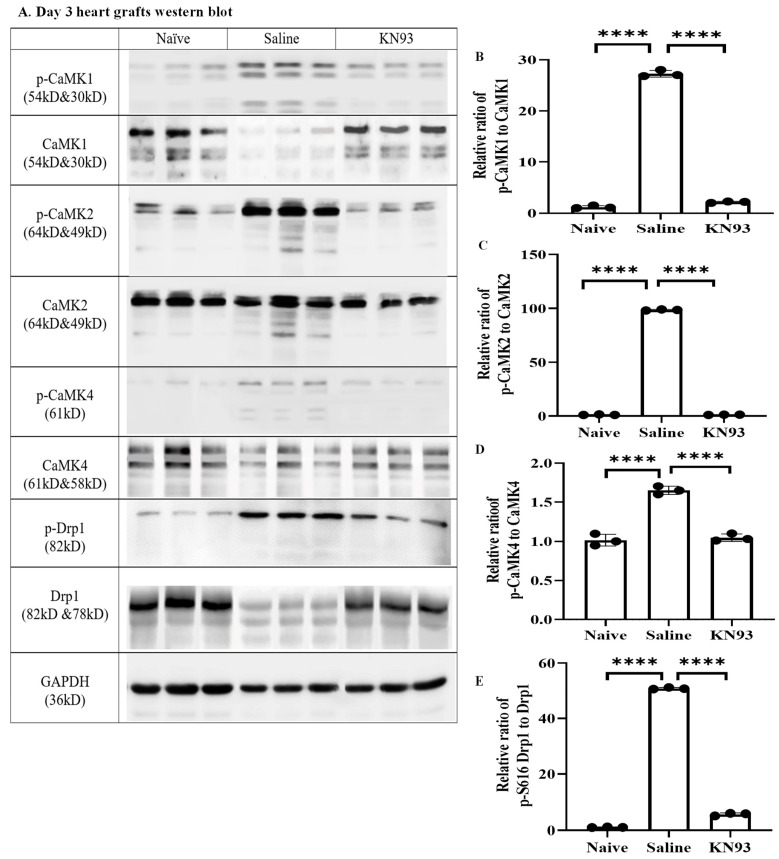
p-CaMK1, p-CaMK2, p-CaMK4, and p-Drp1 (S616) increases were inhibited by KN93 in the graft post heart transplantation. (**A**) B6-to-BALB/c heart transplantation and KN93 injection was performed as detailed in the Methods. The grafts (n = 3) were collected after 3 days for Western blot analysis by CaMK1, p-CaMK1, CaMK2, p-CaMK2, CaMK4, p-CaMK4, Drp1 and p-Drp1 (S616) antibodies, respectively. (**B**–**E**) Images were quantified by ImageJ. Relative ratio of protein = phosphorylated protein/total protein. Data are shown as mean ± SD of 3 transplants. **** *p* ≤ 0.0001; Student *t*-test.

Finally, we investigated if inhibition of CaMKs could attenuate graft injury and improve transplant survival using an established murine heart allotransplant model [9,10,11,12]. Donor hearts from wild type B6 mice were perfused with KN93 (10 μg/mL) and then subjected to ischemic storage as described in Section 4 before being transplanted into fully allogeneic BALB/c mice. Graft recipients received KN93 (20 μg/mouse) or 0.5 mL saline intraperitoneally on day 1, 2, and 3 after transplantation. After three days, grafts were collected for Western blot analyses. Interestingly, p-CaMK1, p-CaMK2, p-CaMK4, and p-Drp1 (S616) were increased in transplanted grafts compared with naïve hearts (Figure 6A–E). However, expression levels of these proteins were significantly inhibited by KN93 treatment (Figure 6A–E).

**Figure 7:**
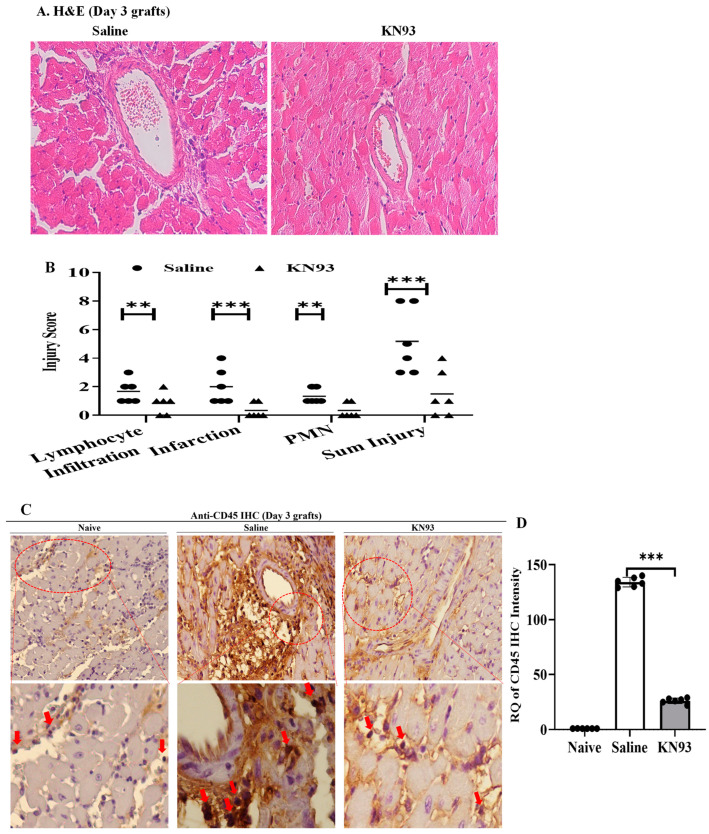

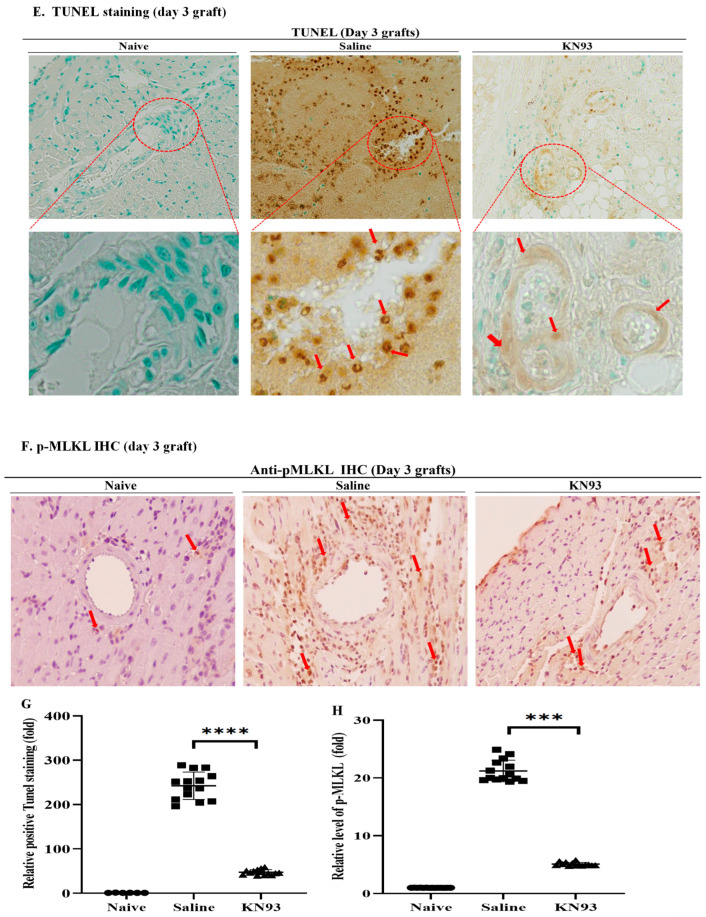
Inhibition of CaMKs attenuates heart transplant acute injury. (**A**) B6-to-BALB/c heart transplantation and KN93 or saline injection were performed as detailed in Section 4. Grafts (n = 6/group) were collected after 3 days for H&E staining. Images were taken under 200 times magnification. (**B**) Graft injuries were scored for lymphocyte infiltration, infarction, and PMN infiltration from 0 to 5 in a blinded fashion. Scores were averaged as mean ± SD of 6 grafts. (**C**) Grafts (n = 6) were used for immunohistochemistry with anti-CD45, and positive areas (brown color) are indicated by red arrows. Images were taken under 200 times magnification. (**D**) Positive areas of each graft were automatically counted in six connected random areas under 200 times magnification by Image J and averaged in a double-blinded manner. (**E**) Grafts (n = 6) were assessed by TUNEL. B6 naive hearts were used as control. Brown color indicates TUNEL positive cells as indicated by red arrows. Images are at 200 times magnification. (**F**) Necroptosis in the graft was detected by p-MLKL immunohistochemistry. Images are at 200 times magnification. Positive cells are indicated by red arrows. (**G**) TUNEL positive areas were quantified as above. (**H**) p-MLKL positive areas were quantified as above. ** *p* ≤ 0.01, *** *p* ≤ 0.001, **** *p* ≤ 0.0001. *t*-test.

Next, we analyzed tissue damage following CaMKs inhibition. Additional grafts (n = 6/group) were collected 3 days after transplantation. Graft sections were stained by hematoxylin and eosin (H&E) and tissue damage was scored by a pathologist in a double-blinded fashion. As shown in Figure 7A,B, the KN93 treatment attenuated lymphocyte infiltration (0.8 ± 0.8 vs. 1.7 ± 0.8 in control, n = 6, *p* = 0.048), infarction (0.3 ± 0.5 vs. 2.0 ± 1.3 in control, n = 6, *p* = 0.007), polymorphonuclear leukocyte (PMN) infiltration (0.3 ± 0.5 vs. 1.3 ± 0.5 in control, n = 6, *p* = 0.003) and overall injury (1.6 ± 1.2 vs. 5.2 ± 2.3, n = 6, *p* = 0.005). Graft lymphocyte infiltration was furthermore confirmed by immunohistochemistry using anti-CD45 (Figure 7C,D). TdT-mediated dUTP nick-ended labeling (TUNEL) indicated the area of cell death in heart grafts was significantly inhibited in KN93 treated recipients (Figure 7E,G). KN93 treatment also reduced p-MLKL levels in the graft (Figure 7F,H), implying its role in inhibiting necroptosis in the graft. 

**Figure 8:**
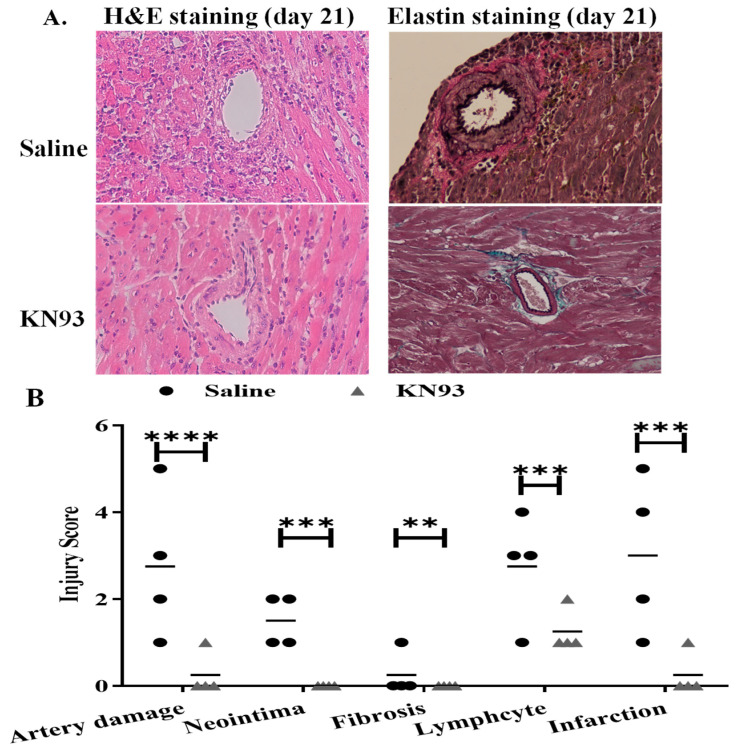

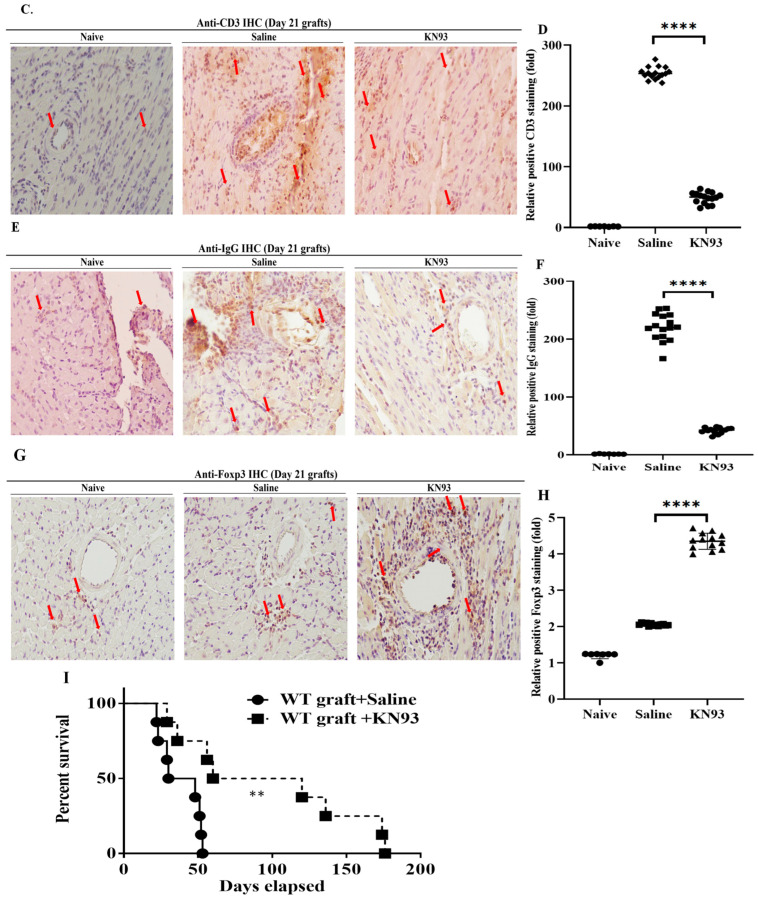
Inhibition of CaMKs attenuates heart transplant chronic injury and rejection. (**A**) B6-to-BALB/c heart transplantation and anti-CD154 injection are detailed in Section 4. KN93 or saline was injected on day 1, 2, and 3 followed by every 48 h until 21 days post transplantation. Recipient mice (n = 4/group) were euthanized, and the grafts were collected for H&E and elastin-trichrome staining. Images were taken under 200 times magnification. Representative images are shown. (**B**) Graft injuries were quantified blindly by a pathologist. Scores were averaged as mean ± SD of 4 grafts. ** *p* ≤ 0.01, *** *p* ≤ 0.001, **** *p* ≤ 0.0001, *t*-test. Grafts were assessed by immunohistochemistry for anti-CD3 (**C**), anti-IgG (**E**) and anti-Foxp3 (**G**) and positive staining areas (brown color) are indicated by red arrows. Images were taken under 200 times magnification. Positive areas anti-CD3 (**D**), anti-IgG (**F**) and anti-FoxP3 (**H**) of each graft were automatically counted in six connected random areas under 200 times magnification by Image J and averaged in a double-blinded fashion. **** *p* ≤ 0.0001. *t*-test. (**I**) B6-to-BALB/c heart transplantation using KN93 or saline administration was performed as above. Graft survival was monitored daily. Cessation of beating is considered as rejection. n = 8 per group, ** *p* = 0.004. Log Rank test.

To determine if KN93 can affect transplant long term and attenuate chronic rejection, B6 hearts were perfused with KN93 solution or vehicle control and subjected to ischemic storage as above. All graft recipients received immunosuppression consisting of anti-CD154 on the day of transplantation. KN93 solution or 0.5 mL saline was administered intraperitoneally for 3 days and then on alternated days for 21 days. The grafts were collected for histological analyses of chronic rejection. A double-blinded evaluation of H&E staining indicated significant inhibition of the microvasculature damage in the KN93 treatment allografts, with low levels of artery damage (0.25 ± 0.5 vs. 2.75 ± 1.7 in control, n = 4, *p* = 0.015), neointima formation (0 vs. 1.5 ± 0.58 in control, n = 4, *p* = 0.001), lymphocyte infiltration (1.25 ± 0.5 vs. 2.75 ± 1.26 in control, n = 4, *p* = 0.034), and infarction (0.25 ± 0.5 vs. 3.0 ± 1.83 in control, n = 4, *p* = 0.014) (Figure 8A,B). KN93 treated grafts showed less infiltration of CD3^+^ T cells (Figure 8C,D) and IgG than did control grafts (Figure 8C–E). Interestingly, KN93 treated grafts showed higher Foxp3 staining (Figure 8C,F), implying the potential role of regulatory T cells in improving graft survival.

Finally, we examined if KN93 could improve graft survival in a murine model of heart allotransplantation. B6 hearts were treated with KN93 as above. Interestingly, KN93 treatment attenuated rejection and significantly prolonged graft survival compared with the controls (mean survival = 98.5 ± 62.6 days vs. 38.5 ± 13.7 days, n = 8, *p* = 0.004, Figure 8G). In summary, these data demonstrate that inhibition of CaMKs attenuated graft IRI and prolonged transplant survival.

## 3. Discussion

We previously reported that RIPK1/3 and mitochondrial molecules participate in necroptosis and transplant rejection [9,10,11,12,14]. However, it has remained unknown how RIPK1 and RIPK3 interact with mitochondria during necroptosis. In this study, we have identified that CaMKs are the intermediary link between RIPK3 and down-stream mitochondrial damage. Furthermore, we have found that the CaMK family members differentially regulate the phosphorylation of the mitochondrial fission molecule, Drp1, during necroptosis. Of clinical relevance, CaMKs inhibition attenuated heart transplant injury and significantly prolonged heart graft survival. Our results strongly suggest that CaMKs play crucial roles in necroptosis and thus might be clinically important targets to prevent transplant injury and rejection.

CaMK1–4 are multifunctional serine/threonine protein kinases and can phosphorylate multiple downstream targets. CaMK2 consists of four homologous subtypes (α, β, γ, and δ). CaMK2α/β are mostly found in neural systems [15]. Interestingly, CaMK2δ is a major type of CaMK2 in heart cells (reviewed in [16,17,18]). Interestingly, a recent study has found that CaMK2 is a substrate of RIPK3 and participates in necroptosis and apoptosis in heart injury [19]. Furthermore, several studies have demonstrated that the RIPK3-CaMKII-mPTP pathway plays an important role in necroptosis and the myocardial pathogenesis (reviewed in [20,21]). Our study supports these findings and clearly demonstrates that RIPK3 is linked to CaMK2 in MVECs during necroptosis (Figure 3). Studies have demonstrated that CaMK2 participates in mitochondrial dysfunction via activation of Drp1 in neuronal and cancer studies [22,23]. However, it was unknown whether CaMK2-mediated-Drp1 activation is a direct or indirect effect. Our study shows that CaMK2 is linked to PGAM5 and thus indirectly activates Drp1 (Figure 5).

CaMK1 and CaMK4 are activated via CaMKK-dependent phosphorylation [24,25,26,27]. They also regulate mitochondrial morphology via activation of Drp1 in neuronal and kidney cells [28,29]. However, it was unclear if CaMK1/4 directly or indirectly activate Drp1. In this study, we have found that CaMK1/4 can directly activate p-Drp1 (S616) in the absence of PGAM5 (Figure 4 and Figure 5). This differs from CaMK2 which requires PGAM5 for Drp1 activation (Figure 5). CaMKs have distinct subcellular distributions after phosphorylation, CaMK1 and CaMK2 execute their functions in the cytosol while CaMK4 locates within the nucleus [16,17]. However, in our immunocytochemistry studies, all CaMKs could be found in the cytosol (Supplementary Figure S1), implying a different role of CaMK4. Indeed, we showed that CaMK4 directly binds to Drp1 during necroptosis (Figure 5), indicating a non-nuclear role of CaMK4.

Drp1 accumulates within mitochondria once activated and drives mitochondrial fission [30,31]. Its excessive activation leads to mitochondrial dysfunction and ultimately cellular apoptosis [32]. Interestingly, Drp1 also plays a role in necroptosis. A previous study suggested that the RIPK1-RIPK3-PGAM5-Drp1 axis drives mitochondrial damage and necroptosis [33]. However, the role of Drp1 in necroptosis remained controversial as divergent studies from several groups had shown that PGAM5 and Drp1 are ‘disposable’ for cellular necroptosis [34,35,36]. We found that Drp1 is activated via two different pathways: the RIPK3-CaMK2-PGAM5-Drp1 axis and the RIPK3-CaMK1/4-Drp1 axis (Figure 4 and Figure 5). This may explain in part why PGAM5 is not required for necroptosis in some studies, as CaMK1/4 can bypass PGAM5 and directly activate Drp1 [34,36]. Interestingly, a recent study showed that inhibition of Drp1 reduced endothelial cells immunogenicity to allogeneic CD8^+^ T cells sufficiently to protect mouse heart allografts from injury and prolong allograft survival [37]. In our transplantation study, inhibition of Drp1 upstream CaMKs significantly inhibited graft injury and prolonged heart graft survival (Figure 7 and Figure 8). It is noteworthy that the activation forms of p-CaMKs and p-Drp1 were increased post transplantation, which could be significantly inhibited by KN93 treatment (Figure 6). CaMKs inhibition in the present transplant study may have multiple effects beside inhibiting necroptosis as CaMKs are multi-targeting kinases and thus participate in other death pathways such as apoptosis [38,39,40,41,42] and pyroptosis [43,44]. Hence, our study cannot eliminate an effect of CaMKs inhibition on other death pathways to improve graft survival. In addition, CaMKII in the heart has multiple variants including δA, δB, δC, and δ9 (Reviewed in [18,21]). While δB protects against cell death, other variants participate in cell death [18,21]. Therapeutic benefit by pharmacological inhibition of this pathway is challenged by the diversity of CaMKII isoforms and splice variants. Effective clinical therapies will need to consider how to best uncouple harmful from beneficial effects of targeting CaMKII. In our study, the results of CaMK inhibitor treatment reflected an overall benefit despite broadly inhibiting CaMKIIδ isoforms. However, selective targeting of specific CaMKII variants of course may provide greater and more predictable protection in cell death and heart transplantation.

Our in vivo results are based on a mouse heterotopic heart transplantation model, which, like other small animal studies, have limitations in extrapolating to potential human clinical studies. Mouse heart transplantation being a heterotopic model is not life supporting as in conventional clinical orthotopic transplantation. Thus, there are differences in heart chamber usage, physiology, and effects on myocardial stress that can influence results. In addition, humans have more complex innate and memory alloimmune responses compared with mice, which as well can affect responses to inflammatory injury and cell death. However, mouse models provide valuable insights that form a basis for translational studies in larger animal models and ultimately clinical studies. Our results thus support further studies in larger animal models.

In summary, our studies confirm that CaMKs and RIPK3 interact to form a complex which is related to mitochondrial damage and necroptosis. Inhibition of CaMKs can impede cell death and can prolong allogenic heart transplant survival, thus supporting the physiological importance and potential clinical relevance of this interaction. This study supports that CaMKs are important in inducing programmed cell death and thus may be important potential therapeutic targets in preventing organ injury.

## 4. Materials and Methods

### 4.1. Animals

Wild-type C57BL/6 (B6) and BALB/c mice (Charles River Lab, Bar Harbor, ME, USA) were maintained in the animal facility. All animal experimental procedures complied with the guideline from Institutional Animal Care and Use Committee (IACUC) and were approved by the Animal Care Committee of Western University (AUP-2019-131).

### 4.2. Cell Death Assay

MVEC were isolated and developed as described previously [9,10]. Cells were grown in DMEM medium supplemented with fetal bovine serum. TNFα (10 ng/mL; PeproTech, Rocky Holl, NJ, USA) was used to induce cell death. Smac-mimetic BV6 (2 μM; ApexBio Tech. Houston, TX, USA) was added to suppress the function of inhibitor of apoptosis proteins (IAP) and therefore activates caspases to promote cell death. Caspase-mediated apoptosis was inhibited by caspase-8 inhibitor IETD (30 μM; ApexBio). RIPK1 inhibitor Nec-1s (10 μM; Millipore Sigma, Etobicoke, ON, Canada) was added to inhibit necroptosis. Cell death was measured by SYTOX^®^ Green (100 nM, Thermo Fisher Scientific, Mississauga, ON, Canada) and quantified using the IncuCyte System (Essen Bioscience).

### 4.3. Mitochondrial Analysis

Mitochondrial function and cell death were confirmed by measurement of total ATP level according to the manufactural protocol (Cell Viability Assay, Promega, Madson, WI, USA). In addition, mitochondria were labelled by MitoTracker probes (Thermo Fisher Scientific). Fluorescent intensity was automatically quantified by the IncuCyte System (Essen Bioscience).

### 4.4. Small Interference RNA (siRNA)

MVECs were seeded at 50–60% confluence and washed with serum-free DMEM. MVECs were transfected with the siRNA working solution containing different concentrations of siRNA, Endofectamine (Thermo Fisher Scientific) in serum-free Opti-MEM (Thermo Fisher Scientific) according to the manufactory protocol.

siRNA sequences are: *CaMK1* sense 5′CCACCCUUUUAUGAUGAAAtt-3′, and anti-sense 5′-UUUCAUCAUAAAAGGGUGGgt-3′; *CaMK*2δ sense 5′-GGAUGGACU UUCACAGAUUtt-3′, and anti-sense 5′-AAUCUG UGAAAGUCCAUCCct-3′; *CaMK*4 sense 5′-GAGAU CCUCUGGGCGAUU U UU-3′, and anti-sense 5′ UCAAGG AAAU AUUCGAAACUU-3′; *PGAM5* sense 5′-CCATAGAGACCACCGATAT-3′, and anti-sense 5′-CGGAA GCTGTGCAGTATTA-3′; *Drp1* sense 5′-CGUAAAAGGUUGCCCGUGAtt-3′, and anti-sense 5′-UCACGGGC AACCUUUUACGaa-3′.

MVECs were transfected with siRNA. siRNA-induced silencing was confirmed by PCR and Western blot analysis. Based on PCR result, the dose of 50 nM siRNA was selected in this study. The cells were harvested at 24-h post-transfection and used for cell death assays.

### 4.5. Quantitative PCR

Total RNA was extracted using TRIzol Reagent (Thermo Fisher Scientific). PCR was performed using PowerTracker QPCR Mix (Thermo Fisher Scientific). The primers used are as follows: *CaMK1*: 5′-CCAGGTGGAAGCAGGCGGAA-3′ and 5′-AGAAGGCAC CCGTGCCCAGA-3′. *CaMK*2δ: 5′-CCTAAATGGCATAGTTCAC-3′ and 5′-GGATCTTTA CGCAGGACTTC-3′. *CaMK*4: 5′-AGGAGACCTCCAGTATGGTGC-3′ and 5′-CTCCTCA GTCATGGGGTCCAT-3′. *Drp*1: 5′-TGGGTGCGGACATCA-3′ and 5′-GCTCTGCGTT CC CACTACGA-3′. PGAM5: 5′-ATCTGGAGAAGACGAGTTGACA-3′ and 5′-CCTGTTCCC GACCTAATGG T-3′. *β*-*actin*: 5′-CCAGCCTTCCTTCCTGGGTA-3′ and 5′-CTAGAACAT T GCGGTGCA-3′. β-actin was used as endogenous control for gene expression analysis.

### 4.6. Immunoprecipitation

Cells were collected in RIPA Lysis and Extraction Buffer containing a protease inhibitor cocktail (Cell Signaling Technology, Danvers, MA, USA). Each supernatant was supplemented with 1 μg of the appropriate antibody and incubated for 1–2 h at 4 °C. An aliquot (20 μL) of protein G agarose (Santa Cruz Biotech, Dallas, TX, USA) was added to each sample and incubated at 4 °C overnight. The beads were then washed. An aliquot (40 μL) of SDS–PAGE sample buffer was added to the beads to elute the immunoprecipitated proteins for Western blot analysis.

### 4.7. Western Blot

Total cellular protein was extracted using RIPA buffer with protease inhibitors. The concentration of the isolated protein was measured by Bradford Dye Protein Assay (Thermo Fisher Scientific). The samples were then equally loaded in 10% SDS-PAGE gel. Antibodies used in Western blot are as follows: anti-CaMK1 (sc-137225), anti-p-CaMK1 (sc-28438-R), anti-CaMK2 (sc-100362), anti-Drp1 Drp1 (sc-271583), anti-GAPDH (sc-47724, Santa Cruz Biotech); anti-p-CaMK2 (PA5-37832), anti-p-CaMK4 (PA5-36745, Thermo Fisher Scientific); anti-CaMK4 (4032S) anti-PGAM5-L (63454S, Cell Signaling Technology); anti-p-Drp1 (S616) (ab314755), and anti-RIPK3 (ab62344, Abcam, Toronto, ON, Canada). Protein bands were visualized by chemiluminescent substrate (Millipore Sigma, Oakville, ON, Canada) and imaged in the FluorChem M Imaging System (Protein-Samples, Ottawa, ON, Canada). Protein was quantified by densitometry (ImageJ 1.54g).

### 4.8. Heterotopic Cardiac Transplantation and Post-Operative Monitoring

Donor hearts from B6 mice were heterotopically transplanted into the abdominal region of BALB/c mice [9]. Mouse was injected with ketamine/xylazine and placed above a heating pad at 37 °C. A median sternotomy was operated. To induce transplantation IRI, the donor heart was perfused with Ringer’s buffer and stored at 37 °C for 1 h and at 4° C for 4 h. Some hearts were perfused with CaMKs inhibitor KN-93 (20 μg/mL, MilliporeSigma). The donor heart was procured through a butterfly thoracic incision and then anastomosed to the recipient abdominal aorta and inferior vena cava using 11-0 sutures (Ethicon, Piscataway, NJ, USA). Following blood reperfusion, the heart graft resumed spontaneous contraction. The vena cava and pulmonary veins in the donor hearts were sutured shut. The recipients were kept on inhaled isoflurane/oxygen mixture (MilliporeSigma). The abdominal wall and skin were closed with a 5-0 suture (Ethicon, Piscataway, NJ, USA.

The graft recipients received KN93 (20 μg/mouse) or 0.5 mL saline injection intraperitoneally on day 1, 2, and 3, and then every other day until day 21. Anti-CD154 (0.25 mg/mouse, 740874, BD Biosciences, San Jose, CA, USA) was injected intraperitoneally right after transplant surgery. Graft survival was monitored by abdominal palpation for pulse detection. Cessation of or a significant drop in cardiac pulsation was considered as graft rejection that is confirmed by histopathological analysis.

### 4.9. Histology

Grafts were collected and perfused with saline, cut transversely, then fixed with 5% formalin. Paraffin sections were used for H&E and elastin staining. All injury scores of artery damage, infarction, and leukocyte infiltration were evaluated by a pathologist in a blind manner. Damage was scored on a scale of 0–5 (0: no change, 1: 0–10% change, 2: 10–25% change, 3: 25–50% change, 4: 50–75% change, 5: >75% changes).

Tissue sections were stained by anti-CD3 (ab243874), anti-CD45 (ab10558), anti-IgG (ab238004), and anti-FoxP3 (ab238004, Abcam) followed by immunohistochemistry. Intragraft death in tissue sections was detected by TUNEL method (MilliporeSigma). The number of TUNEL-positive cells were automatically quantified by Image J in a double-blinded fashion.

### 4.10. Statistical Analyses

Data were analyzed using the Student’s *t*-test or 1-way ANOVA with Tukey’s post-hoc corrections test. The Mantel–Cox log rank test was used to determine graft survival differences. Differences were considered significant when the *p*-value was ≤ 0.05.

## 5. Conclusions

Our in vitro and in vivo studies confirmed that CaMKs and RIPK3 form a complex and subsequently mediate mitochondrial damage and necroptosis. Importantly, inhibition of CaMKs can prevent cells death and organ injury and prolong allogenic heart transplant survival. Hence, our study indicates that CaMKs are important cell death inducers and thus potential therapeutic targets in preventing organ injury.

## Data Availability

The data presented in this study are available on request from the corresponding author.

## Supplementary Materials

The following supporting information can be downloaded at: https://www.mdpi.com/article/10.3390/ijms25084428/s1.

## Abbreviations

CaMK	calmodulin-dependent protein kinase
Drp1	dynamin-related protein-1
IRI	Ischemia reperfusion injury
MVEC	mouse microvascular endothelial cells
Nec-1s	Necrostatin-1
PCR	polymerase chain reaction
PGAM5	phosphoglycerate mutase 5
pMLKL	phosphorylated mixed lineage kinase domain like protein
RIPK	receptor interacting protein kinase
TUNEL	TdT-mediated dUTP nick-ended labeling
Z-IETD	Z-Ile-Glu-Thr-Asp-Fluoromethylketoe
