# Supplementary figures and images for: The CaMK Family Differentially Promotes Necroptosis and Mouse Cardiac Graft Injury and Rejection
Source: Int J Mol Sci. 2024 Apr 17;25(8):4428. doi: 10.3390/ijms25084428 (PMC11050252; doi:10.3390/ijms25084428)

Supplemental Figure S1

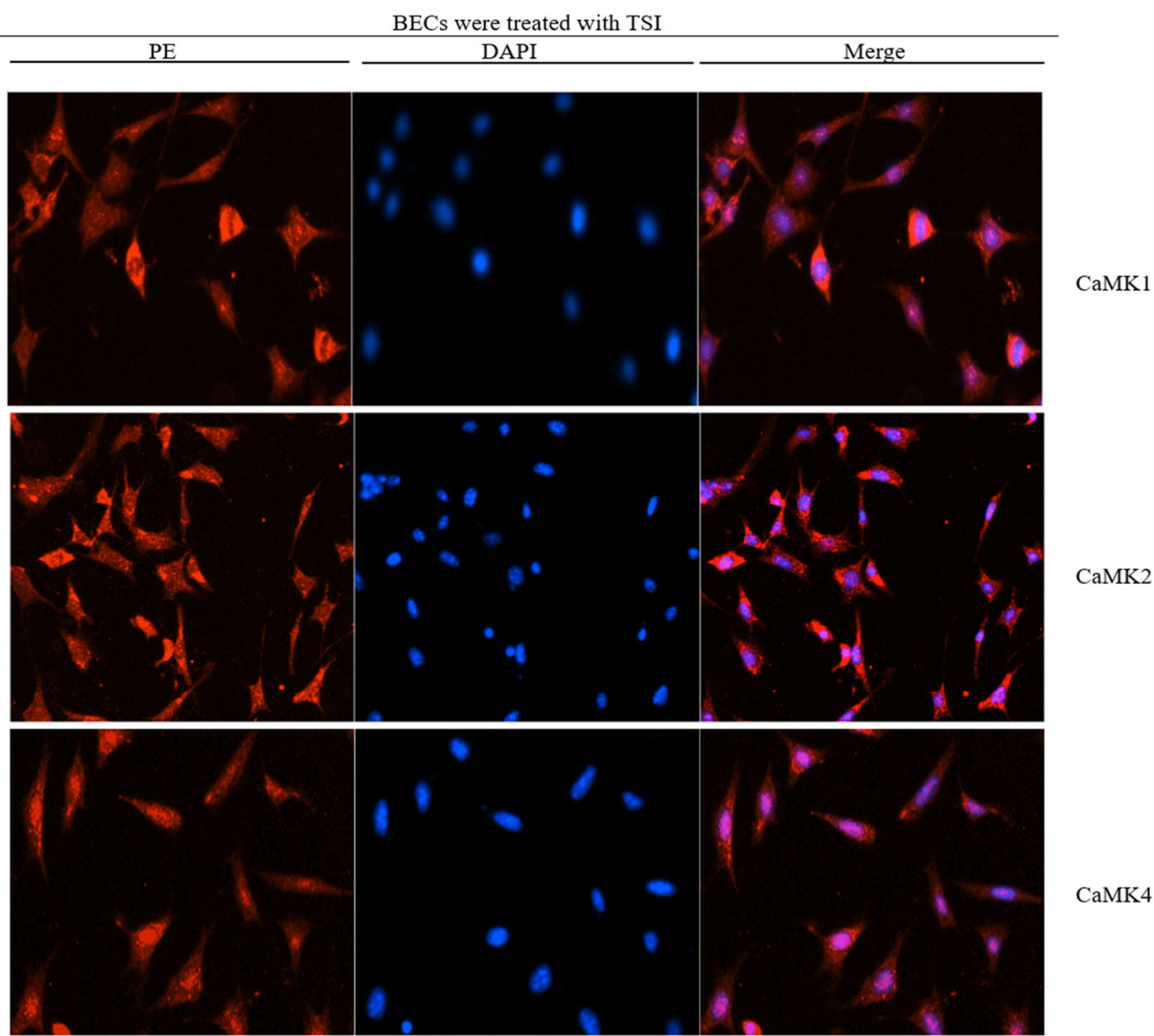

Supplement: Supplementary file 1 [file ijms-25-04428-s001.zip › ijms-2855660-supplementary.pdf]
